# Evaluation of the effects of hesperidin on fresh and frozen-thawed semen quality using two different cryopreservation methods in Simmental bull

**DOI:** 10.1590/1984-3143-AR2022-0042

**Published:** 2022-10-11

**Authors:** Hamid Tahmasbian, Esmail Ayen, Amir Khaki

**Affiliations:** 1 Urmia University, Urmia, West Azerbaijan, Iran; 2 Amol University of Special Modern Technologies, Amol, Mazandaran, Iran

**Keywords:** hesperidin, semen freezing method, sperm characteristics, Simmental

## Abstract

In the industry of bull semen freezing centers, one-step and two-step semen dilution protocols are two standard and well-known methods in semen freezing process. As the freezing/thawing processes cause detrimental effects on sperm function, the addition of antioxidants can improve sperm characteristics. Hesperidin (Hesp) is an antioxidant used as the male reproductive protective agent. Therefore, the aim of this study was to investigate two different dilution methods, as well as to evaluate Hesp supplementation influence on sperm characteristics in fresh and frozen thawed semen. Semen samples were collected from 12 Simmental bulls. Two separate examinations were conducted in, with and without Hesp supplementation groups. Statistical analysis was performed by an independent t-test, Mann Whitny test, MANOVA and ANOVA tests. In comparison to the one and two-step dilution protocols without Hesp supplementation, the two-step dilution showed greater cryoprotective potential. In the Hesp supplemented group, each semen sample was divided into six equal parts for experimental groups (dilution step method/µM of Hesp). In the both one and two step dilution protocols, significant improvements were detected in semen motility parameters by Hesp administration. Also, oxidative stress status was reduced in seminal plasma of Hesp treatment groups. Interestingly, in comparison with Hesp dosage, 1µM was shown to have greater semen cryoprotective potential. In conclusion,

## Introduction

Different methods of freezing have been used for bulls' semen, including slow and fast freezing. Slow freezing is more common because of less expensive protocol and better semen handling, but heat transfer in semen is too slow, which could induce crystallization in sperm cells (Barbas and Mascarenhas, 2009; Arav et al., 2002). Besides, one-step dilution (semen packaging at room temperature) and two-step dilution (semen packaging at +4 °C) methods are used as two main procedures for freezing bull semen in AI stations. However, each dilution method has its own pros and cons (Buranaamnuay, 2017).

In one-step dilution, minimal manipulation with less contamination and time consumption is suggested for straw production. Also, all semen processing stages, including packaging, equilibrating, and freezing are performed after semen mixing, which reduce sperm shock in many species (García-Álvarez et al., 2010). However, the two-step dilution presented less glycerol toxicity in the cooling and equilibration stage of sperm freezing, thereby minimizing its harmful influence (Brito et al., 2017; Peña and Linde-Forsberg, 2000). In two-step dilution, glycerol addition was performed in the second stage of dilution, which showed better cryoprotective potential than one and three-step dilution methods in dog, bulls, and rams (Peña and Linde-Forsberg, 2000; Arif et al., 2020; Colas, 1975). But, one-step dilution showed better cryoprotective effect on rams’ semen compared to two-step dilution, which could indicate that glycerol concentration was not the only issue for semen protection (Jha et al., 2019). However, some other studies indicated no significant differences between the one and two-step dilution methods (Stănescu and Bîrţoiu, 2012).

Hesperidin (Hesp) as the main flavanone derivative is detected in Citrus fruits, such as tangerines, oranges, limes, and lemons. It is a white needle-like powder with a molecular weight of 610 and C28H34O15 chemical formula (Peterson et al., 2006; Lanza, 2003). For the first time, it was found in orange peel and lemons. It was then introduced to the industry as an inexpensive byproduct (Evans, 2009; Zhang and Demain, 2005). Because of the Hesp wide range of pharmacological effects, various variants, such as Glucosyl- Hesp (G- Hesp) (Kometani et al., 2008; Yamada et al., 2006), Hesp -7,3'-O-dimethylether (HDME) (Ko et al., 2004) and Hesp methyl chalcone (Chanal et al., 1981; Walle, 2009) were produced. Hesp was used as the skin and wound healer (Kawaguchi et al., 1997; Sa’Ayinzat et al., 2021), anticancer, antioxidant, and anti-inflammatory agent (Aggarwal et al., 2020).

Recent studies suggested Hesp as an antioxidant affected by scavenging reactive oxygen species (ROS) with different mechanisms in the main organelles (Arafa et al., 2009; Pari et al., 2014; Suarez et al., 1998). Antioxidative activity of Hesp was detected in liver (Mahmoud, 2014), kidney (Ahmad et al., 2012), central nerval (de Andrade Teles et al., 2018), cardiovascular (Bunbupha et al., 2021; Zanwar et al., 2014) and reproductive system (Aksu et al., 2021; Afolabi et al., 2019). It has been reported that oxidative stress induced various malfunctions and deformities in male and female reproductive system, especially in sperm parameters in bulls (Morrell, 2020). Besides, significant correlation had been suggested between antioxidant status and frozen-thawed semen quality (El-Khawagah et al., 2020). The role of Hesp in reproductive system was investigated in recent research, which indicated Hesp positive influence on both male and female fertility not only by ROS inhibition but also by hormonal stabilizing, including estrogens, androgens and thyroid hormone.

The protective effect of Hesp in male reproductive system was suggested based on recent research. Methotrexate exposed male rats were ameliorated by 200 mg/kg of Hesp administration for seven consecutive days. Sperm parameters and the level of oxidative enzymes (i.e., CAT, GPx and SOD) decreased and the regular histopathological structure of testis was improved (Belhan et al., 2017). Also, Hesp protective influence was indicated against endocrine disrupting chemicals (phthalate), cancer therapy drugs (Finasteride), varicocele and diabetes in male reproductive system by glucose stabilization, oxidative stress limitation and hormonal regulatory effects (Helmy et al., 2020; Olayinka and Adewole, 2021; Shokoohi et al., 2020; Nerdy et al., 2021). A limited study was conducted for evaluation of Hesp as a cryopreservation agent or its direct effect on fresh semen. A study was conducted for assessing the phosphorylated Hesp effect on fresh semen, which indicated antifertility properties (Chang and Pincus, 1953). To the best of our knowledge, no study has been conducted for evaluation of Hesp influence on semen quality after supplementation to the extender medium for freezing of animal or human fresh and frozen- thawed semen. The current study discusses Hesp as a cryopreservation agent and its role in frozen-thawed semen parameters, as well as antioxidant status in Simmental bulls.

## Methods

### Chemicals

All chemicals used in the current study were produced by Sigma Aldrich (St. Louis, MO, USA), while the other materials were purchased from other companies, as mentioned in the article.

### Preparation of Hesp different dosages

Hesp (97% purity; Sigma-Aldrich Co., St. Louis, Missouri, USA) stock solution was purchased to prepare different Hesp doses. Accordingly, different Hesp concentrations were evaluated to choose 1 and 2 µM of it (data are not published). The 1 µM stock solution was prepared by dissolving 0.61056 g of Hesp in 1mL of 5% Dimethyl sulfoxide (DMSO). Then, 100 µL of the stock solution was mixed with 900 µL distilled water (100 µM Hesp solution). Finally, 10 and 20 µL of 100 µM Hesp solution were dissolved with 90 and 80 µL pre-extender sperm solution, respectively, and 1 and 2 µM Hesp solutions were prepared (Ali et al., 2021; Valipour et al., 2021). Hesp supplementation was performed according to the one- and two-step dilution protocols separately.

### Animals

The Animal Ethics Committee of the Amol University of Modern Technologies approved the animals selected for this study (No: AUEC120). The semen collection was carried out within four months from the end of December 2020 to mid-April 2021. Twelve healthy breeding bulls of the Simmental breed, aged 4-7 years old, were used for this research. The samples were taken during routine weekly semen collection at the Iran Simmental Cattle Breeding Center (elevation: 47 m, longitude: 52˚ 23′57.76′′ E, and latitude: 36˚ 30′ 18.55′′ N) between 8 and 12 AM. Animals were fed three times daily with the formula mentioned in Table 1. The mean humidity and temperature of the study period were 57.42 ±3.62 and 9.37 ± 3.73, respectively.

**Table 1:** Compositions and amounts of *Simmental* bulls’ daily meal.

**Ingredient**	**Amount**		**Chemical composition**				
	**kg**	**Crude protein (%)**	**NDF (%)**	**ADF (%)**	**FAT (%)**	**Ash (%)**	**Dry matter (%)**
Concentrate mixa	9	14.68	16.8	13.1	3.2	7.4	89.2
Silage	18	8.5	54.5	32.7	1.8	5.7	25
Hay	3	16.8	44.7	34.6	2.5	9.7	1.88
Straw	*Ad libitum*	3.9	70.3	45.5	1.1	9.8	94.8
Mineral supplementb	*Ad libitum*						
Water	*Ad libitum*						

NDF: neutral detergent fiber; ADF: acid detergent fiber; FAT: fat; Ash: total mineral content of a diet. ^a^Ca 0.74%, P 0.53%, Na 0.49%, Mg 0.29%, Zn 375 ppm, Mn 381.44 ppm, Co 1.01 ppm, Se 2.75 and vitamin additives (vitamin A 7500 U/kg, vitamin D3 1000 U/kg, vitamin E 10 mg/kg); ^b^Mg 2.1%, Na 7%, Fe 355 mg/kg, Zn 1560 Mg/kg, Cu 390 mg/kg, Mn 1560 mg/kg, Se 7.5 mg/kg, Co 3 mg/kg, I 15.5 mg/kg. Data calculated by animal nutrition laboratory, Faculty of Agriculture, University of Tehran, Iran.

### Semen collection and experimental groups

Semen samples from each bull were collected by pre-warmed artificial vagina at 46 °C in an oven (three repetitions for each cow). The sexual preparation of bulls was performed with three false mounts by standing for 10 min in the collection area. Semen concentration was measured using an SDM photometer (Minitube, Tiefenbach, Germany) calibrated for bull sperm cell counting. To estimate the fresh sperm motility, two small drops from diluted semen were put on a glass slide and analyzed using a binocular phase contrast microscope (Minitube, Tiefenbach, Germany) at a magnification of ×200 equipped with a warm stage. Each ejaculation was evenly divided into six parts for the experimental groups: group 1 (one-step method/control), group 2 (one-step method/1 µM of Hesp), group 3 (one-step method/2 µM of Hesp), group 4 (two-step method/ control), group 5 (two-step method/1 µM of Hesp), and group 6 (two-step method/2 µM of Hesp).

### Preparation of the extender, pre-extender dilution, and final extender volume

The current study used a Steridyl extender (Steridyl, REF: 13500/0260, Minitube, Tiefenbach, Germany) with a composition of TRIS, citric acid, sugar, buffers, glycerol, purest water, irradiated sterile egg yolk, and antibiotics (Tylosin, Gentamicin, Spectinomycin, Lincomycin). A bottle of 500 mL Steridyl and 750 mL double distilled pure water was initially put in a 32-34 °C pre-warmed water bath for 10 minutes to prepare the ready-to-use extender. The distilled water was then added to the extender slowly, which was filled and emptied twice, after which the solution was slowly mixed by a magnetic stirrer, and the pre-extender dilution was prepared by gently adding the extender to the semen with a ratio of 1:1. The solution was then placed in a water bath at 34 °C for 10 min. Finally, the extender volume was calculated based on the following formula: Number of doses = (semen volume × semen concentration × progressive motile sperm × morphologically normal sperm) ÷ (sperm per dose [15 million]).

### Semen freezing

#### The one-step dilution method

The final solution was prepared by adding the pre-extender to the calculated diluent volume. The flasks were placed in a plastic container with a constant level of water from the water bath at 32 °C and then left at room temperature (20 °C) for 15 min. After that, the diluted semen was packed in 0.5 mL straws (Minitube, Slovakia) with the MPP Uno automated filling and sealing machine (Minitube, Tiefenbach, Germany). The packed semen was equilibrated by placing straws in a refrigerator set at 4 °C for 3 hours. Semen, packed in French straws, was frozen in an MT freezing device (Minitube, Tiefenbach, Germany) based on the following protocol: from +4 °C to −12 °C at the rate of −4 °C /min, from −12 °C to −40 °C at the rate of −40 °C /min, and from −40 °C to −140 °C at the rate of −50 °C /min. The frozen straws were then stored in separate goblets within the canisters of the liquid nitrogen container.

#### The two-step dilution method

Before preparing the final diluted semen, the pre-extender left at 32 °C for 10 min was slowly added to the bottle, which contained 20 mL of the prepared steridyl solution and was pre-warmed in a water bath at 32 °C. This solution was placed in a plastic container filled with water from the water-bath at 32 °C. The bottle was then placed in a cold cabinet at +4 °C for 1.5 hours. The final diluent was then prepared by adding the remaining calculated diluent already kept in the cold cabinet at +4 °C. The diluted semen was equilibrated for 5 hours away from light in the cold cabinet. The diluted semen was packed in 0.5-ml straws (Minitube, Slovakia) at +4 °C in a cold cabinet with the MPP Uno automated filling and sealing machine (Minitube, Tiefenbach, Germany). Then the straws were placed on racks. The freezing protocol was the same for both methods.

### Assessment of the sperm motility, viability, morphology, and membrane integrity

**Figure 1:**
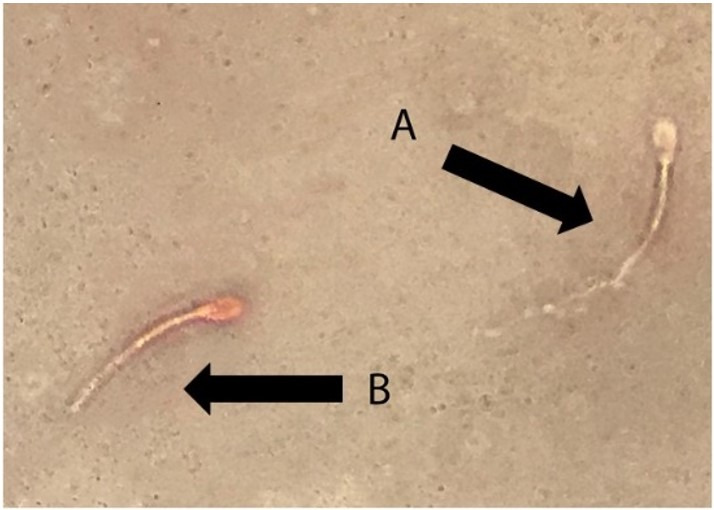
Eosin nigrosin staining of Simmental semen. (A) viable sperm and (B) dead sperm (×1000 magnification).

**Figure 2:**
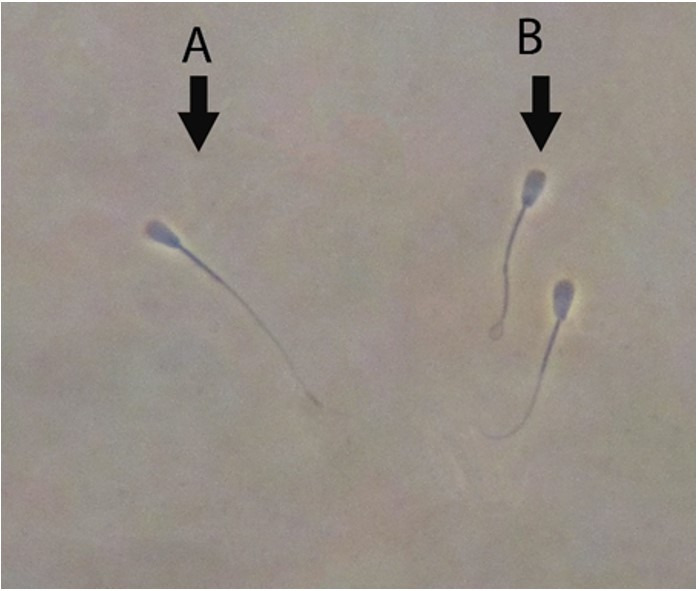
HOST assessment technique for the functional integrity of the sperm membrane. (A) HOST negative (non-coiled) and (B) HOST positive (coiled) Simmental sperm (×1000 magnification).

The mobility of both fresh and frozen-thawed sperms was determined using CASA software (Hooshmand Fanavar, Tehran, Iran). The straws were put in a water bath adjusted at 37 °C for 40 s to thaw the frozen semen. Parameters, such as progressive motility (PM), curvilinear velocity (VCL), straight-line velocity (VSL), average path velocity (VAP), degrees of deviation (MAD), lateral head displacement (ALH), beat cross frequency (BCF), linearity (LIN [VSL/VCL]), wobble (WOB [VAP/VCL]), and straightness (STR [VSL/VAP]) were evaluated. All analyses were performed using the light microscope equipped with a hot plate that maintained samples at 37 °C and also a chamber (Sperm meter, Depth 10-micron, Surface Graticule, 100x 0.1 SQMM) to avoid the decline in the sperm motility during the analysis. Eosin-nigrosine (Minitube, Tiefenbach, Germany) staining was used to assess viability and morphological abnormality by examining 200 sperms per sample at 400 x magnification (Figure 1). Hypoosmotic swelling test (HOST) has been suggested as one of the critical tests for sperm membrane functional integrity. The swollen and curled sperm tails changed their shape during the examination, and flagellar swelling caused various coiled or swollen tails (Figure 2). The HOST was conducted by the addition of 50 μL of the semen sample (fresh or frozen-thawed) to 450 μL of the hypo-osmotic solution (prepared from tri-sodium citrate dihydrate and fructose) (Ijaz et al., 2009). The glass slide was preheated, and two drops of the final solution were placed on and covered with a coverslip. At least 200 sperms were counted by a binocular phase contrast microscope (Minitube), and the percentages of sperms with curled tails were calculated.

### The total antioxidant capacity (TAC)

TAC was measured using a kit (Naxifer™ -Cat# NS-15012; Total Antioxidant Capacity Assay Kit-TAC; Navand Salamat Company; Urmia; Iran) in seminal plasma based on manufacture protocols. The diluted seminal plasma (1:10) was gradually mixed with stock solutions. The TAC content in the final solution was then detected by a spectrophotometer (Thermo Fisher Scientific; Waltham. MA, USA) at 593 nm (Khaki et al., 2019).

### Lipid’s peroxidation (MDA)

The seminal plasma MDA status was detected according to the thiobarbituric acid (TBA) protocol. Straws were thawed as described before, and semen was centrifuged twice for 10 min at 3000 rpm. Subsequently, the TBA reagent was added to samples based on the kit protocol, and the final solution was read by a spectrophotometer at 586 nm (Dwinofanto et al., 2019).

### Statistical analysis

All collected data were expressed as the mean ± S.D when comparing the one and two-step dilutions without Hesp supplementation. The independent t-test and Mann-Whitney were used for parametric and non-parametric data. In the Hesp supplemented groups, the difference between the one and two-dilation steps, as well as the protective effect of different doses of Hesp on semen parameters and oxidative status, were evaluated using the two-way multivariate analysis of variance (MANOVA) and one-way analysis of variance (ANOVA) test using SPSS software (Version 23.0; SPSS, Chicago, USA). P-Value < 0.05 was considered for significance.

## Results

In the current study, semen quality in the one and two-step dilution methods was evaluated with and without different doses of Hesp supplementation. A significant difference was observed between the Hesp different dosage groups (Pillai's T = 0.900, F =3.589 and P = 0.00) and different dilution protocol groups (Pillai's T = 0.454, F =3.634 and P = 0.00). However, no significant difference was detected in Dilution type × Hesperidin Treatment (Pillai's T = 0.215, F =0.530 and P = 0.999).

As significant differences were detected between the one and two-step dilution methods, including viability before freezing (VBF), mid piece sperm abnormality before freezing (Mid.pBF), cytoplasmic droplet before freezing (Cy.DrBF) and progressive motility of frozen thawed semen (PMFT) (Table 2), a separate analysis was performed on each group. In the one step-dilution group, 1st Hesep/1µM group, PM before freezing (PMBF), VSL before freezing (VSLBF), LIN before freezing (LINBF), WOB before freezing (WOBBF) and STR before freezing (STRBF) parameters increased significantly, whereas BCF before freezing (BCFBF) parameter decreased significantly compared to 1step/control group (P<0.05). Additionally, in the 1 step /2 µM group, LIN before freezing (LINBF), WOB before freezing (WOBBF) and STR before freezing (STRBF) parameters increased significantly, whereas BCFBF and BCF of frozen thawed semen (BCFFT) parameters decreased significantly compared to the 1step/control group (P<0.05). Moreover, in both 1 step/1 and 2 µM groups, Hesp significantly reduced TAC status in frozen thawed semen (Table 3).

**Table 2:** Effect of one and two step dilution method on semen characteristics (36 semen samples). Data are represented as the mean ± standard deviation (Mean ± SD).

**Parameters**	**1 Step (Means ± SD)**	**2 Step (Means ± SD)**	**Sig**
**PMBF (%)**	75.2950 ± 6.23758	74.4306 ± 8.07180	0.613
**VBF (%)**	67.1536 ± 9.94669	70.1436 ± 8.97427	0.011
**Mid.pBF (%)**	0.8847 ± 0.78048	1.4556 ± 1.28854	0.021
**Cy.DrBF(%)**	0.2283 ± 0.33942	0	0
**MADFT (%)**	16.0547 ± 7.51018	19.4722 ± 9.20680	0.121
**HOSTBF (%)**	53.828 ± 11.962	44.0958 ± 13.866	0.002
**PMFT (%)**	53.8883 ± 11.44779	59.1369 ± 10.59799	0.047
**VFT (%)**	67.1536 ± 9.94669	70.1436 ± 8.97427	0.185
**HOSTFT (%)**	30.4161 ± 9.79536	33.7825 ± 11.25614	0.18
**TAC**	0.6132 ± 0.08751	0.6095 ± 0.08185	0.853

PMBF: Progressive motility before freezing; VBF: Viability before freezing; Mid.PBF: Mid peace before freezing; Cy.DrBF: Cytoplasmic Droplet before freezing; MADFT: Degrees of deviation frozen-thawed; HOSTBF: Hypoosmotic swelling test before freezing; PMFT: Progressive motility frozen-thawed; VFT: Viability frozen-thawed; HOSTFT: Hypoosmotic swelling test frozen-thawed; TAC: Total antioxidant capacity.

**Table 3:** Effect of different concentrations of hesperidin on Simmental semen characteristics (36 semen samples). Data are represented as the mean ± standard deviation (Mean ± SD).

**Analysis**	**Dilution**	**Control**	**Hesp (1Micro)**	**Hesp (2Micro)**
**PMBF (%)**	One step	68.7820 ± 6.95312^Ab^	75.9928 ± 6.18381^Aa^	72.3736 ± 6.37069^Ab^
	two step	65.9200 ± 7.02956^Ab^	72.8242 ± 8.20401^Aba^	70.9419 ± 7.45606^Aa^
**VCLBF (μm/S)**	One step	48.9507 ± 12.05783^Aa^	50.7017 ± 11.42826^Aa^	46.4623 ± 14.02948^Aa^
	two step	44.8761 ± 14.02017^Aa^	47.5947 ± 14.20815^Aa^	45.6397 ± 13.70373^Aa^
**VSLBF(μm/S)**	One step	20.1777 ±6.43296^Ab^	23.4329 ± 4.89550 ^Aa^	20.8191 ± 5.90696^Ab^
	two step	19.2033 ± 6.92307^Aa^	22.5177 ± 6.14258^Aa^	20.6376 ± 5.04294^Aa^
**VAPBF (μm/S)**	One step	28.8122 ± 7.39711^Aa^	31.0513 ± 6.73986^Aa^	28.1929 ±7.86176^Aa^
	two step	27.0347 ± 8.30843^Aa^	29.5772 ± 8.44571^Aa^	27.9742 ± 7.41388^Aa^
**MADBF**	One step	26.8525 ± 9.11947^Aa^	25.5819 ± 9.18562^Aa^	23.6406 ± 9.96291^Aa^
	two step	23.4948 ± 10.29157^Aa^	24.0709 ± 10.01270^Aa^	22.5422 ± 9.25050^Aa^
**ALHBF (%)**	One step	2.8910 ± 0.60483^Aa^	2.6441 ± 0.66132^Aa^	2.5146 ± 0.75762^Aa^
	two step	2.6125 ± 0.71028^Aa^	2.4651 ± 0.65265^Aa^	2.5141 ± 0.72040^Aa^
**BCFBF (Hz)**	One step	2.1358 ± 0.98454^Aa^	1.0894 ± 1.15301^Ab^	1.0914 ± 1.1358^Ab^
	two step	1.8642 ±1.26322^Aa^	1.0844 ± 1.12616^Ab^	1.2092 ± 1.19158^Aa^
**LINBF (%)**	One step	38.0005 ± 5.91696^Ab^	43.9118 ±4.76987^Aa^	42.2219 ±5.24958^Aa^
	two step	37.0942 ± 5.24979^Ab^	41.9044 ± 8.97310^Aa^	41.4436 ± 5.98330^Aa^
**WOBBF**	One step	55.4797 ± 4.82374^Ab^	59.7186 ± 3.87961^Aa^	58.3797 ± 4.59558^Aa^
	two step	54.2544 ± 5.19605^Ab^	58.6492 ± 5.56108^Aa^	57.4864 ± 4.88458^Aa^
**STRBF (%)**	One step	57.7394 ± 6.91087^Ab^	65.6947 ± 5.16779^Aa^	62.3206 ± 6.46422^a^
	two step	56.0122 ± 6.86032^Ab^	63.4083 ± 7.29329^Aa^	60.9269 ± 7.37807^Aa^
**PMFT (%)**	One step	53.1300 ± 10.36457^Aa^	57.2639 ± 13.04017^Aa^	56.8406 ± 11.51512^Aa^
	two step	53.5706 ± 8.80864^Ab^	59.3806 ± 8.88674^Aa^	54.0081 ± 10.77536^ABb^
**VCLFT (μm/S)**	One step	33.4619 ± 11.83573^Aa^	34.2418 ± 13.26563^Aa^	31.8632 ± 12.04218^Aa^
	two step	35.2605 ± 10.32654^Aa^	35.1933 ± 9.28471^Aba^	31.3577 ± 11.40506^Aa^
**VSLFT (μm/S)**	One step	14.1758 ± 6.54778^Aa^	14.5761 ± 6.40390^Aa^	14.1051 ± 6.26444^Aa^
	two step	13.8111 ± 4.27865^Aa^	15.1792 ± 5.07590^Aa^	12.8511 ± 4.57821^Aa^
**VAPFT (μm/S)**	One step	20.0454 ± 6.98319^Aa^	20.6552 ± 8.41401^Aa^	19.3951 ± 7.72459^Aa^
	two step	20.3221 6/02124^Aa^	20.5647 5/11255^Aa^	18.1279± 6.27029^Aa^
**MADFT**	One step	17.0938 ± 7.73901^Aba^	15.7589 ± 7.65132^Aba^	14.3866 ± 6.88173^Aa^
	two step	18.7681 ± 6.72034^Aa^	17.3600 ± 6.36957^Aa^	15.3999 ± 7.56416^Aa^
**ALHFT (%)**	One step	2.1539 ± 0.65113^Aa^	2.0070 ± 0.61639^Aa^	1.8860 ± 0.60872^Aa^
	two step	2.1518 ± 0.61062^Aa^	2.0033 ± 0.53018^Aa^	1.7876 ± 0.54151^Ab^
**BCFFT (Hz)**	One step	1.2134 ± 0.67951^Aa^	0.6441 ± 0.69020^Aa^	0.4711 ± 0.72523^Ab^
	two step	1.2022 ± 0.99218^Aa^	0.4583 ± 0.53910^Aa^	0.4261 ± 0.56437^Ab^
**LINFT (%)**	One step	31.5581 ± 8.53227^Aa^	35.0130 ± 8.59829^Aa^	35.6910 ± 7.62878^Aa^
	two step	30.6356 ± 5.40931^Ab^	35.3731 ± 5.81933^Aa^	32.1736 ± 7.08870^Ab^
**WOBFT**	One step	47.2397 ± 9.54965^Aa^	49.9608 ± 10.09219^Aa^	49.6619 ± 9.11402^Aa^
	two step	46.2817 ± 6.84280^Aa^	49.5314 ± 6.39216^Aa^	46.0344 ± 8.23561^Aa^
**STRFT (%)**	One step	46.8536 ± 11.09669^Aa^	51.3989 ± 12.96305^Aa^	50.6392 ± 11.19694^Aa^
	two step	47.2342 ± 7.73150^Aa^	51.5436 ± 11.77694^Aa^	47.5534 ± 9.55090^Aba^
**TAC (mmol/L)**	One step	0.4687 ± 0.07613^Aa^	0.3367 ± 0.10196^Ab^	0.2937 ± 0.09357^Ab^
	two step	0.4954 ± 0.08064^Aa^	0.3410 ± 0.09997^Ab^	0.2907 ± 0.06924^Ab^
**MDA (mmol/L)**	One step	0.2664 ± 0.06202^Aa^	0.2551 ± 0.04168^Aa^	0.2573 ± 0.04156^Aa^
	two step	0.2667 ± 0.03330^Aa^	0.2471 ± 0.03632^Aa^	0.2533 ± 0.04070^Aa^
**AMBF (%)**	One step	19.0556 ± 12.14089^Aa^	19.8026 ± 12.78697^Aa^	19.3629 ± 11.68925^Aa^
	two step	19.9771 ± 13.23370^Aa^	18.2301 ± 11.95642^Aa^	19.3779 ±12.04810^Aa^
**VBF (%)**	One step	75.8153 ± 7.46274^b^	81.8592 ± 4.90198^Aa^	79.2278 ± 7.25332^Ab^
	two step	74.5411 ± 7.67684^Ab^	79.2375 ± 5.86175^Aa^	78.1608 ± 8.51778^Ab^
**HOSTBF (%)**	One step	46.4556 ± 14.34913^Aa^	46.5646 ± 13.61395^Aa^	50.3725 ± 12.38123^Aa^
	two step	40.1086 ± 13.49428^ABa^	43.2075 ± 12.40066^Aba^	40.1081 ± 11.99943^Ba^
**AMFT (%)**	One step	20.6831 ± 12.27241^Aa^	19.2450 ± 11.15419^Aa^	19.6403 ± 11.73932^Aa^
	two step	20.1335 ± 12.42494^Aa^	18.3231 ± 11.38895^Aa^	19.3072 ± 12.26062^Aa^
**VFT (%)**	One step	61.4761 ± 9.69590^Aa^	61.0258 ± 15.25005^Ba^	62.2544 ± 9.65218^Aa^
	two step	61.8842 ± 8.77109^Aa^	65.2761 ± 9.15060^Aa^	61.8989 ± 9.40200^Aa^
**HOSTFT (%)**	One step	30.3753 ± 11.22396^Aa^	26.6839 ± 9.72286^Aa^	27.9926 ± 8.12691^Aa^
	two step	29.1217 ± 10.32546^Aa^	27.8756 ± 10.71020^Aa^	27.8444 ± 10.00463^Aa^

Different letters (a,b) indicate significant differences between the data in each row (p<0.05) . Different letters (A,B) indicate significant differences between the data in each column (p<0.05). PMBF: Progressive motility before freezing; VCLBF: Curvilinear velocity before freezing; VSLBF: Straight-line velocity before freezing; VAPBF: Average path velocity before freezing; MADBF: Degrees of deviation before freezing; ALHBF: Lateral head displacement before freezing; BCFBF: Beat cross frequency before freezing; LINBF: Linearity before freezing; WOBBF: VAP/VCL before freezing; STRBF: VSL/VAP before freezing; PMFT: Progressive motility frozen-thawed; VCLFT: Curvilinear velocity frozen-thawed; VSLFT: Straight-line velocity frozen-thawed; VAPFT: Average path velocity frozen-thawed; MADFT: Degrees of deviation frozen-thawed; ALHFT: Lateral head displacement frozen-thawed; BCFFT: Beat cross frequency frozen-thawed; LINFT: Linearity frozen-thawed; WOBFT: VAP/VCL frozen-thawed; STRFT: VSL/VAP frozen-thawed; TAC: Total antioxidant capacity; MDA: Malondialdehyde; AMBF: Abnormal morphology before freezing; VBF: Viability before freezing; HOSTBF: Hypoosmotic swelling test before freezing; AMFT: Abnormal morphology frozen-thawed; VFT: Viability frozen-thawed; HOSTFT: Hypoosmotic swelling test frozen-thawed.

In the two-step dilution method/ 1 µM of Hesp and 2 µM of Hesp groups, PMBF, LINBF, WOBBF, STRBF, PMFT, VBF and LIN of frozen thawed semen (LINFT) increased compared to control group, but BCFBF, BCFFT, ALH of frozen thawed semen (ALHFT) and TAC reduced significantly (Table 3).

In comparison between Hesp doses, the 1 µM Hesp solution group indicated better protective influence compared to 2 µM Hesp solution. Significant alteration was only indicated between 1 µM Hesp solution and control group in PMBF, VSLBF, VBF and BCFFT in the one-step dilution method and in BCFBF, VBF, PMFT, ALHFT, BCFFT, and LINFT parameters in the two-step dilution method (Table 3).

Correlations between all semen parameters were evaluated by Spearman’s correlation coefficients. In comparison between fresh semen CASA system sperm parameters, a significant positive correlation was observed between PMBF, VCL of frozen thawed semen (VCLBF), VSLBF, VAP before freezing (VAPBF), MAD before freezing (MADBF), ALH before freezing (ALHBF), BCFBF, LINBF, WOBBF, STRBF, VBF, and HOST, as well as between each of the above mentioned parameters. A negative correlation was shown between the mentioned parameters and MADBF, sperm head abnormal morphology before freezing (HeadBF), Mid.pBF, Cy.DrBF and abnormal morphology of sperm tail before freezing (TailBF). In frozen-thawed straws, sperm parameters, including PMFT, VCLFT, VSL of frozen thawed semen (VSLFT), VAP of frozen thawed semen (VAPFT), MAD of frozen thawed semen (MADFT), ALHFT, BCFFT, LINFT, WOBFT, STR of frozen thawed semen (STRFT), viability of frozen thawed semen (VFT) and HOST of frozen thawed semen (HOSTFT) were positively correlated with each other. A negative correlation was also indicated between sperm abnormal morphology of frozen thawed semen (MorphFT), sperm head amnormal morphology of frozen thawed semen (HeadFT), mid piece sperm abnormality of frozen thawed semen (Mid.PFT), cytoplasmic droplet of frozen thawed semen (Cy.DrFT), sperm tail abnormal morphology of frozen thawed semen (TailFT) and the mentioned parameters in frozen-thawed semen. The negative correlation between oxidant parameters and semen characteristics was only detected between TAC and PMBF, VSLBF, LINBF WOBBF, STRBF, VBF and HOST. Interestingly, a positive correlation was detected between BCFBF and BCFFT, and TAC (Table 4).

**Table 4:** Correlations coefficient between sperm parameters and antioxidant level in Simmental seminal plasma.

	**PMBT**	**VCLBT**	**VSLBT**	**VAPBF**	**MADBF**	**ALHBF**	**BCFBF**	**LINBF**	**WOBBF**	**STRBF**	**VBF**	**MABF**	**HeadBF**	**Mid.PBF**	**Cy.DrBF**	**TailBF**	**HOSTBF**	**PMFT**	**VCLFT**	**VSLFT**	**VAPFT**	**MADFT**	**ALHFT**	**BCFFT**	**LINFT**	**WOBFT**	**STRFT**	**ViabFT**	**MAFT**	**HeadFT**	**Mid.PFT**	**Cy.DrFT**	**TailFT**	**HOSTFT**	**TAC**	**MDA**
PMBF	1	.537**	.751**	.639**	.387**	.209**	-.012	.841**	.830**	.954**	.619**	-.271**	-.281**	-.087	.109	-.167*	.184**	.286**	.257**	.275**	.270**	.218**	.201**	.111	.267**	.271**	.257**	.255**	-.274**	-.256**	-.126	-.037	-.248**	.142*	-.286**	-.051
VCLBF		1	.887**	.967**	.961**	.899**	.748**	.236**	.412**	.520**	.499**	-.313**	-.391**	-.135*	.155*	**-.113**	.184**	.200**	.276**	.246**	.262**	.268**	.284**	.276**	.130	.167*	.174*	.313**	-.249**	-.295**	-.176**	.113	-.163*	.217**	-.027	.051
VSLBF			1	.966**	.752**	.656**	.486**	.591**	.705**	.765**	.576**	-.350**	-.396**	-.135*	.122	-.161*	.140*	.201**	.250**	.236**	.241**	.230**	.224**	.188**	.155*	.171*	.173*	.291**	-.307**	-.318**	-.191**	.051	-.261**	.155*	-.159*	-.007
VAPBF				1	.876**	.814**	.665**	.405**	.575**	.629**	.548**	-.352**	-.412**	-.148*	.128	-.144*	.168*	.197**	.270**	.242**	.255**	.257**	.267**	.246**	.132	.164*	.167*	.307**	-.293**	-.322**	-.202**	.097	-.223**	.189**	-.097	.011
MADBF					1	.930**	.818**	.032	.216**	.355**	.407**	-.258**	-.345**	-.128	.164*	-.060	.199**	.188**	.268**	.238**	.256**	.265**	.290**	.295**	.120	.161*	.168*	.296**	-.183**	-.244**	-.135*	.118	-.080	.243**	.038	.068
ALHBF						1	.926**	-.119	.124	.154*	.337**	-.275**	-.352**	-.115	.114	-.080	.166*	.119	.216**	.179**	.201**	.214**	.255**	.283**	.049	**.098**	**.100**	.261**	-.204**	-.264**	-.174*	.162*	-.100	.209**	.094	.060
BCFBF							1	-.311**	-.045	**-.080**	.217**	-.208**	-.286**	-.107	.081	-.022	.143*	.042	.160*	**.114**	.141*	.165*	.213**	.261**	-.027	**.031**	**.034**	.187**	**-.132**	-.206**	-.126	.165*	-.018	.207**	.175*	.052
LINBF								1	.905**	.895**	.477**	-.219**	-.194**	-.081	.034	-.163*	.044	.171*	.146*	.165*	.155*	.111	.086	-.005	.181**	.163*	.154*	.143*	-.233**	-.188**	-.083	-.111	-.268**	-.011	-.302**	-.087
WOBBF									1	.849**	.535**	-.270**	-.256**	-.112	.027	-.166*	.079	.166*	.153*	.165*	.162*	.116	.121	.043	.157*	.154*	.136*	.171*	-.272**	-.238**	-.134	-.059	-.280**	-.010	-.327**	-.096
STRBF										1	.592**	-.272**	-.280**	-.095	.124	-.169*	.133	.248**	.230**	.243**	.237**	.197**	.172*	.088	.232**	.225**	.222**	.221**	-.270**	-.250**	-.097	-.072	-.256**	.091	-.298**	-.058
ViabBF											1	-.535**	-.565**	-.254**	.116	-.296**	.331**	.306**	.340**	.320**	.334**	.308**	.314**	.226**	.231**	.280**	.255**	.398**	-.518**	-.517**	-.286**	.163*	-.363**	.158*	-.236**	.016
AMBF												1	.927**	.517**	-.074	.711**	-.234**	-.350**	-.427**	-.425**	-.434**	-.391**	-.381**	-.337**	-.283**	-.351**	-.339**	-.510**	.841**	.838**	.475**	-.139*	.593**	-.176**	.057	.027
HeadBF													1	.491**	-.143*	.496**	-.248**	-.301**	-.379**	-.376**	-.384**	-.357**	-.337**	-.306**	-.239**	-.294**	-.284**	-.483**	.788**	.845**	.445**	-.163*	.474**	-.236**	.021	.013
Mid.PBF														1	-.105	.224**	-.175**	-.186**	-.255**	-.249**	-.255**	-.238**	-.246**	-.218**	-.174*	-.223**	-.209**	-.257**	.414**	.433**	.406**	-.035	.210**	**-.013**	.040	.134
Cy.DrBF															1	-.112	.211**	.150*	.062	.118	.095	.026	.015	**-.038**	.194**	.178**	.157*	.161*	-.081	-.129	-.032	.135*	.017	.174*	-.109	-.119
TailBF																1	-.096	-.321**	-.348**	-.350**	-.352**	-.315**	-.292**	-.240**	-.268**	-.327**	-.319**	-.388**	.611**	.521**	.324**	-.076	.600**	**-.046**	.059	.070
HOSTBF																	1	.388**	.388**	.434**	.424**	.291**	.331**	.223**	.381**	.404**	.393**	.371**	-.259**	-.254**	-.116	.013	-.117	.370**	-.151*	.077
PM.FT																		1	.824**	.916**	.886**	.674**	.636**	.352**	.913**	.957**	.952**	.837**	-.415**	-.359**	-.278**	.012	-.420**	.272**	-.049	.027
VCLFT																			1	.927**	.977**	.951**	.921**	.718**	.643**	.792**	.814**	.762**	-.477**	-.435**	-.303**	.068	-.437**	.325**	.077	.006
VSLFT																				1	.978**	.802**	.750**	.519**	.842**	.912**	.917**	.823**	-.474**	-.430**	-.295**	.049	-.448**	.295**	.052	.021
VAPFT																					1	.884**	.861**	.651**	.753**	.876**	.877**	.801**	-.480**	-.436**	-.301**	.057	-.445**	.322**	.074	.024
MADFT																						1	.927**	.783**	.448**	.622**	.664**	.656**	-.444**	-.405**	-.304**	.077	-.409**	.309**	.116	-.011
ALHFT																							1	.885**	.382**	.603**	.600**	.600**	-.398**	-.365**	-.241**	.088	-.344**	.321**	.127	.022
BCFFT																								1	.077	.318**	.299**	.411**	-.314**	-.308**	-.170*	.082	-.239**	.277**	.254**	.077
LINFT																									1	.951**	.936**	.740**	-.355**	-.297**	-.242**	-.025	-.365**	.209**	-.044	.017
WOBFT																										1	.963**	.789**	-.420**	-.356**	-.282**	.001	-.417**	.255**	.005	.018
STRFT																											1	.791**	-.417**	-.355**	-.277**	.021	-.416**	.267**	-.007	.022
ViabFT																												1	-.581**	-.555**	-.358**	.092	-.512**	.304**	-.005	.064
MorphFT																													1	.924**	.585**	-.087	.768**	-.176**	.079	.000
HeadFT																														1	.519**	-.164*	.536**	-.200**	.073	.008
Mid.PFT																															1	-.133	.392**	-.057	.069	.063
Cy.DrFT																																1	-.022	.043	-.031	-.024
TailFT																																	1	-.079	.070	-.012
HOSTFT																																		1	.036	.008
TAC																																			1	.100
MDA																																				1

PMBF: Progressive motility before freezing; VCLBF: Curvilinear velocity before freezing; VSLBF: Straight-line velocity before freezing; VAPBF: Average path velocity before freezing; LINBF: Linearity before freezing; ALHBF: Lateral head displacement before freezing; BCFBF: Beat cross frequency before freezing; MADBF: Degrees of deviation before freezing; VBF: Viability before freezing; AMBF: Abnormal morphology before freezing; HeadBF: Head before freezing; Mid.PBF: Mid peace before freezing; Cy.DrBF: Cytoplasmic Droplet before freezing; TailBF: Tail before freezing; HOSTBF: Hypoosmotic swelling test before freezing; PMFT: Progressive motility frozen-thawed; VAPFT: Average path velocity frozen-thawed; VCLFT: Curvilinear velocity frozen-thawed; VSLFT: Straight-line velocity frozen-thawed; VAPFT: Average path velocity frozen-thawed; LINFT: Linearity frozen-thawed; ALHFT: Lateral head displacement frozen-thawed; BCFFT: Beat cross frequency frozen-thawed; MADFT: Degrees of deviation frozen-thawed; VFT: Viability frozen-thawed; AMFT: Abnormal morphology frozen-thawed; HeadFT: Head frozen-thawed; Mid.PFT: Mid peace frozen-thawed; Cy.DrFT: Cytoplasmic Droplet frozen-thawed; TailFT: Tail frozen-thawed; HOSTFT: Hypoosmotic swelling test frozen-thawed; TAC: Total antioxidant capacity; MDA: Malondialdehyde. *95% significant; **%99 significant.

## Discussion

The current study showed the effects of different doses of Hesp and two different semen freezing methods on Simmental bulls’ sperm characteristics and oxidative stress status. Different cryopreservation methods have been designed to achieve a higher quality of frozen-thawed semen (Arif et al., 2020; Jha et al., 2019). The comparison of the one and two-step dilution protocols showed less toxicity for sperm in the two-step dilution because of the slower exposure of sperm to glycerol (Brito et al., 2017); On the other hand, less contamination and easy handling of the one-step dilution method increased this protocol usage in AI stations (Schulze et al., 2018).

In comparison between different freezing methods, the Bangladeshi ram semen was evaluated in the one-, two-, and three-step dilutions. Fresh semen evaluations revealed no significant alterations in none of the diluent groups, including sperm viability and motility parameters, as well as the sperm plasma membrane and acrosome integrity (Jha et al., 2019). However, according to the assessment of the frozen-thawed semen, the two- and three-step dilution groups showed higher sperm viability and motility percentage compared to the one-step dilution method. There were no significant differences between the two- and three-step dilutions in Sperm motility, viability, and plasma membrane integrity parameters (Jha et al., 2019). Additionally, the Dog Spermatozoa assessment indicated that the two-step method was better than the one-step (Peña, and Linde-Forsberg, 2000; van den Berghe et al., 2018). The Holstein-Fresian bulls semen assessment revealed the better cryoprotectivity of the one-step than the two-step dilution method in the fresh and post-thawed semen CASA evaluation (Uavechanichkul et al., 2010). Our study indicated that the two-step dilution protocol improved sperm characteristics, viability, and membrane integrity in Simmental bulls. Also, the positive influence of the two-step dilution protocol was shown on oxidative stress parameters.

Regarding the beneficial effect of the two-step dilution protocol on sperm quality, there were no significant alterations between the one and the two-step dilution protocols in semen samples collected from five mature Merino (Salamon, 1968), five mature Dorset-Polled, and one Hampshire ram (Morrier et al., 2002). On the other hand, the one-, two-, and three-step dilutions were examined for oxidative stress parameters, CASA-based sperm characteristics, and semen quality. Based on the results, there were no significant alterations between the one- and the two-step dilution methods, but higher MDA and AST levels were detected in the three-step compared to the one-step dilution and higher MDA and AST levels in the one- and three-step compared to the two-step dilution. The sperm plasma membrane integrity could not be evaluated perfectly, but there was a significant reduction between the one- and three-step dilutions (Arif et al., 2020). Moreover, fresh boar semen was collected from three European AI centers to assess the one- and two-step dilution methods. The thermal-resistance test indicated significantly higher semen quality in the one- compared to the two-step methods. However, frozen-thawed sperm characteristics were not evaluated by computer or human visual-based assessment (Schulze et al., 2018).

The protective potential of Hesp has been suggested in semen and the male reproductive system as supplementary material by alleviating oxidative stress responses, inhibiting lipid accumulation and obesity without any toxic effects even at high dosages for an extended period of administration (Shen et al., 2019; Shin et al., 2011; Hozayen, 2012). In vivo administration of Hesp ameliorates the toxic effects of various subjects, including vanadium, γ-radiation, and heat stress (Vijaya Bharathi et al., 2015; Shaban et al., 2017; Saeed et al., 2019). Limited research revealed the cryoprotective potential of Hesp, especially as semen pre-extender supplementation, which was evaluated in the current study. Sperm characteristics, plasma integrity, and viability examination indicated that Hesp dosage improved significantly compared to the control group. In addition, oxidative stress decreased in Hesp treatment groups. There was also a significant improvement in the sperm quality (motility, viability and antioxidant status) after administrating 1µM Hesp compared to 2µM Hesp usage. The 1µM Hesp dosage was added to the extender as the proper dosage for a marked enhancement in the quality of the frozen–thawed Simmental spermatozoa. Interestingly, the protective effects of Hesp on the frozen-thawed semen of Simmental bulls indicated by the current study complement the findings by Valipour et al. (2021), who studied the cryoprotective effects of Hesperidin, the aglycone form of Hesp on normozoospermic men. Pre-extending of 20 µM Hesperidin before straw freezing improved the frozen-thawed human semen quality. The CASA system-based evaluation showed that the motility characteristics of diluted sperm/Hesp were significantly better than the control group. Moreover, there was a significant alteration in the following parameters: Viability, morphology, apoptotic-like changes, and oxidative stress markers, which revealed the cryoprotective potential of Hesp on human semen (Valipour et al., 2021).

Besides, quercetin (Q), a plant pigment flavanol with a structure similar to Hesp, has been identified to have potential sperm cryoprotective effects on buffalo, rooster, canine, stallion, and bulls (Najafi et al., 2020; Kawasaki et al., 2020; Gibb et al., 2013; Tironi et al., 2019). Holstein bulls’ semen collected to examine different Q concentrations (25, 50, 100, and 200 μg/ml) significantly affected oxidative stress and frozen-thawed sperm parameters. A similar freezing protocol was employed in all groups with the Tris-based extender. Frozen-thawed sperm characteristics were analyzed using the CASA system, and a significant alteration was detected between all the treatment and control groups. Sperm plasma membrane integrity, abnormality, viability, DNA integrity, and oxidative stress parameters were improved significantly by Q in a dose-dependent manner (Avdatek et al., 2018). Also, a similar dose-dependent cryoprotective manner of Q was detected in Egyptian Buffalo bulls, and the level of 10 µM Q in the OptiXcell extender was suggested as the best antioxidative concentration based on the post-thawed semen quality examination (El-Khawagah et al., 2020). A recent study evaluating the antioxidative effect of Q on ten adult Holstein Friesian breeding bulls’ semen imposed with ferrous ascorbate showed that this polyphenol could protect semen quality by the assessment of fresh semen oxidative status and CASA system (Tvrdá et al., 2016). Altogether, based on the recent studies and our research, the cryoprotective potential of flavanonols, especially Hesp, was suggested, which could be used in commercial semen extenders to improve the frozen-thawed sperm quality.

## Conclusion

According to the results of the present study, bulls’ semen freezing in the two-step dilution method is preferable to the one-step, which might be related to the gradual exposure of spermatozoa to glycerol in semen extender. Moreover, adding 1 µM Hesp to bull semen diluents can improve the quality parameters of the bulls’ semen after thawing by contributing as a beneficial antioxidant. It can also prevent oxidative stress and effectively protect spermatozoa membranes, as reflected by the assessment of the semen parameters. Considering all parameters, Hesp can be used in the semen extenders due to its positive influence on the frozen-thawed semen, while its combination with the two-step dilution protocol can improve the frozen-thawed sperm characteristics in bulls. It is noteworthy that the current research is the first study that compares the effects of two different semen freezing methods in combination with antioxidant supplementation on the extender. Further investigation is suggested to detect the molecular and exact mechanisms of Hesp supplementation, as well as different semen freezing protocols separately or together on the frozen-thawed semen quality. Morover, further research is required to evaluate the effects of Hesp on the fertilizing potential of sperm cells cryopreserved with Hesp supplemented in the extender.
